# An Evolving Inquiry of Monastic Spiritual Care for Aging Inmates

**DOI:** 10.1093/geroni/igab046.510

**Published:** 2021-12-17

**Authors:** Alex Bishop, Kevin Randall

**Affiliations:** 1 Oklahoma State University, Oklahoma State University, Oklahoma, United States; 2 Sam Houston State University, Sam Houston State University, Texas, United States

## Abstract

This investigation involved focus-group inquiry of the Oblates in Prison Program, a faith-based ministry founded on monastic principles in the Rule of St. Benedict. Data from a Benedictine Order monk and program coordinator, ordained prison minister, and lay ministry volunteer were collected. Participants were asked a series of questions regarding the spiritual care of aging prisoners. Responses were coded and cross-compared for thematic content. Of central thematic importance was implementation of a spiritual care model using traditional monastic rules for daily living. A second theme centered on purposeful rebuilding of self-renewal through stability and obedience. A final emergent theme encompassed institutional acceptance in the provision of religious sacraments, sacred texts, and artifacts. Results highlight the broader implications of providing spiritual care and outreach to aging prisoners. The role of restorative justice for successful delivery of faith-based spiritual care for improved rehabilitation of aging inmates will be further addressed.

